# Serpentine Supravenous Hyperpigmentation, a Phenomenon Following the Administration of Chemotherapeutic Agents: A Systematic Review

**DOI:** 10.1002/hsr2.70294

**Published:** 2024-12-19

**Authors:** Hanieh Radkhah, Saba Maleki, Razman Arabzadeh Bahri

**Affiliations:** ^1^ Internal Medicine Department, School of Medicine, Sina Hospital Tehran University of Medical Sciences Tehran Iran; ^2^ School of Medicine Guilan University of Medical Sciences (GUMS) Rasht Guilan Province Iran; ^3^ School of Medicine Tehran University of Medical Sciences Tehran Iran

**Keywords:** chemotherapy, docetaxel, serpentine supravenous hyperpigmentation

## Abstract

**Background:**

Serpentine supravenous hyperpigmentation (SSH) is known as a phenomenon occurring during the infusion of chemotherapy agents in the underlying veins. Chemotherapy agents have potential to cause infusion reactions when used systematically. Linear hyperpigmentation and reticular hyperpigmentation are the differential diagnosis for this phenomenon. The aim of the present study was to systematically review the serpentine supravenous dermatitis induced by chemotherapeutic agents.

**Methods:**

A comprehensive search was conducted in Scopus, PubMed, Embase, and Web of Science bibliometric databases on February 7, 2023. The search keywords were categorized into two groups: SSH and chemotherapy. Any combination between keywords was used for the systematic search. We included any type of article that evaluated the SSH in cancer patients after the infusion of chemotherapeutic agents, including observational studies with at least one eligible patient based on our criteria, case series, and case reports. Studies that reported SSH in non‐cancer patients or caused by any medications other than chemotherapeutic agents were excluded.

**Results:**

Twenty‐five studies were included based on our inclusion criteria consisting of 26 patients. A total of 13 different cancers were reported in the included studies. Lung cancer was the most reported cancer. Also, the mostly reported region of this dermatitis was forearm which was reported in 13 studies. Docetaxel has been used in a total of 11 articles in this study and has independently induced serpentine supravenous dermatitis in seven studies, which is the mostly reported chemotherapeutic agent resulting into serpentine supravenous dermatitis. Most of these skin lesions were self‐limiting and with a normal histopathological finding.

**Conclusion:**

SSH is a dermatologic reaction, which mostly occur when there is peripheral venous access for the injection of chemotherapeutic agents. The skin lesion will improve spontaneously and have a benign course with no abnormal histopathological pathological finding.

## 1 Introduction

Chemotherapy agents have potential to cause infusion reactions when used systematically [1]. Platinum drugs like cisplatin, carboplatin, and oxaliplatin can cause urticaria, pruritus, angioedema, and other symptoms of anaphylaxis due to type 1 immunoglobulin E (IgE)‐mediated allergic reactions [2]. Cutaneous vasculitis due to the administration of methotrexate and serum sickness following the infusion of rituximab can occur through Type 3 immune reaction [3, 4, 5]. Pigmentary changes in nails, hair, and skin can be observed in patients receiving cytotoxic drugs like vincristine, fluorouracil, vinorelbine, fotemustine, bromodeoxyuridine, docetaxel, bleomycin, and combination regimens [6].

Serpentine supravenous hyperpigmentation (SSH) is known as a phenomenon occurring during the infusion of chemotherapy agents in the underlying vein [7]. This phenomenon was reported as the side effect of 5‐fluorouracil (5‐FU) administration by Hrushesky [8]. There is a supravenous pigmentary pattern that is common in the use of 5‐FU, fotemustine, vincristine, vinorelbine, and docetaxel [9, 10].

**Figure 1:**
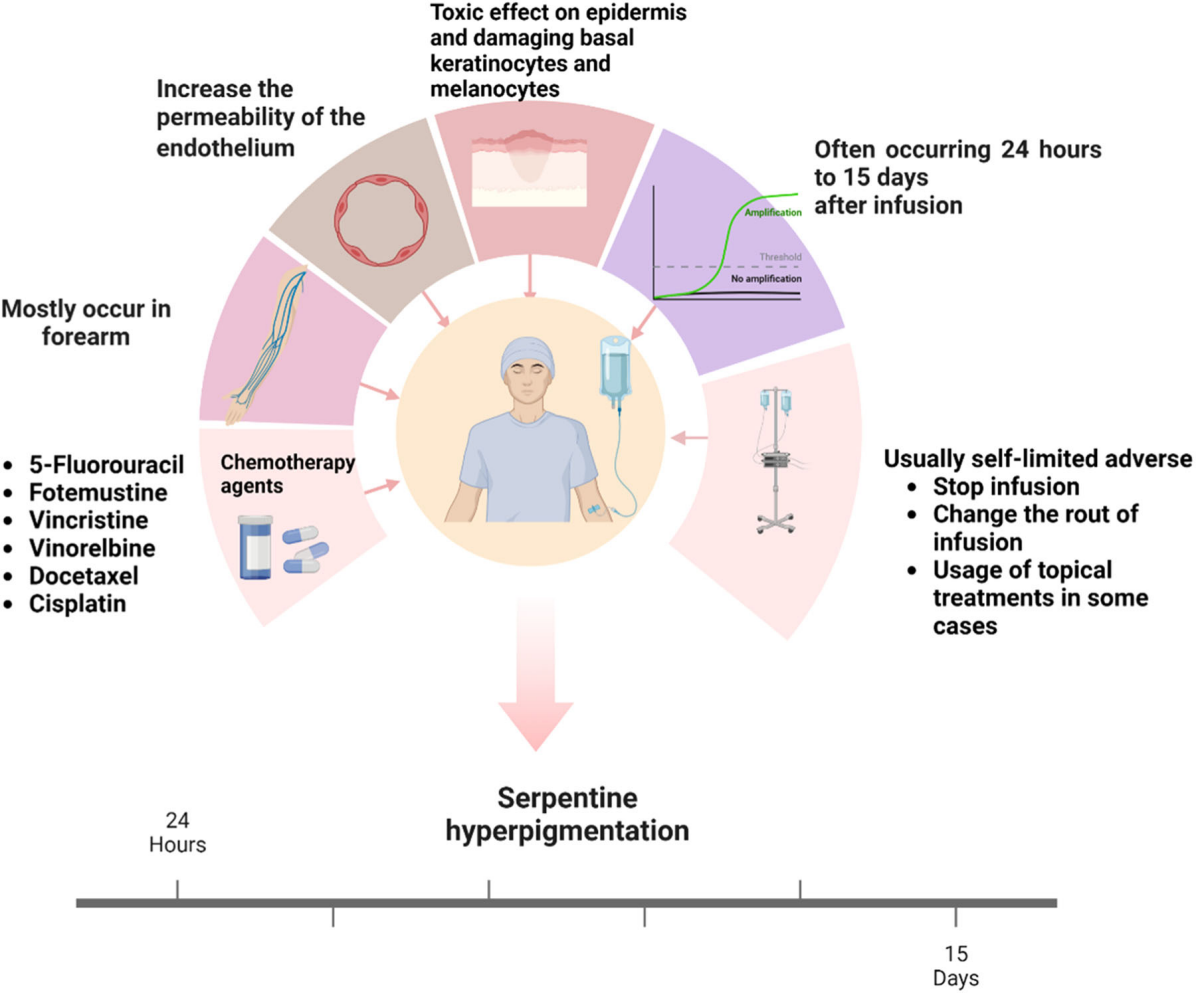
Summarized information of serpentine hyperpigmentation.

This phenomenon usually appears after 24 h until 15 days of chemotherapy agent infusion [11]. This condition usually was seen on the forearm and less common happens in pheripheral areas such as back, femoral, hip, and popliteal fossa [7]. These lesions on the affected region would be disappeared once the infusion stopped [7, 9]. This is known as a self‐limited disease. The pathophysiology of this phenomenon is unknown, but the cytotoxicity of the chemotherapy drugs may increase the permeability of the veins, and the penetration of drugs can cause a toxic effect on the epidermis [12]. Linear hyperpigmentation and reticular hyperpigmentation are the differential diagnosis for SSH. Linear hyperpigmentation is usually seen due to administration of bleomycin through the trauma to the skin or scratching the infusion site [13, 14]. Reticular hyperpigmentation mostly occur on the trunk and lower extremities during the administration of paclitaxel, cytarabine, fuorouracil, and idarubicin [15, 16] (Figure 1).

This systematic review was conducted on the purpose of gathering data regarding the different aspects of this phenomenon from manifestations to diagnosis and treatment based on all case reports reported until now to aid and prevent clinicians from unnecessary treatments.

## 2 Methods

The present systematic literature review was conducted to systematically review the studies that assessed and reported the chemotherapeutic agents‐induced SSH on cancer patients. The methods used for this study were based on the Preferred Reporting Items for Systematic Reviews and Meta‐Analyses (PRISMA) and the Cochrane book.

### 2.1 Search Strategy

We have conducted a comprehensive search in Scopus, PubMed, Embase, and Web of Science bibliometric databases on February 7, 2023. We imposed no limitations on publication time or the main language of the articles. The search keywords were categorized into two groups: SSH and chemotherapy. In the SSH group, we used any possible keywords such as SSH, superficial venous hyperpigmentation, and SSH. In the chemotherapy group, we used all possible keywords, including chemotherapy, chemotherapeutics, docetaxel, paclitaxel, fluorouracil, 5‐FU, vinorelbine, and cisplatin. The keywords were combined with “OR” in each group and with “AND” between the groups.

### 2.2 Eligibility Criteria

We included any type of article that evaluated the SSH in cancer patients after the infusion of chemotherapeutic agents, including observational studies with at least one eligible patient based on our criteria, case series, and case reports. We also included letter to editor articles that reported cases of cancer patients who presented SSH during their course of chemotherapy. We excluded the studies that reported SSH in non‐cancer patients or caused by any medications other than chemotherapeutic agents.

### 2.3 Data Extraction and Quality Assessment

The initial screening of the studies was conducted by two independent reviewers based on their titles and abstracts to exclude non‐related studies. Then, the full texts of the remained articles were reviewed for confirmation of the inclusion criteria and extraction of their data. The studies with similar demographic status were evaluated by a third reviewer to assess the eligibility status of those articles. The data extraction was carried out using an Excel‐based sheet by two reviewers. The data sheet contained the name of the first authors, year of publication, gender and age of the patients, type of the cancer of the patients, the main treatment for cancer, duration of the treatment, start date of the dermatologic reaction, region of the dermatologic reaction, tool for confirmation of the final diagnosis, treatment for the lesions, and the outcome of the patients regarding the dermatologic reaction. The methodological quality assessment of the included studies was performed using JBI critical appraisal tool by two independent reviewers (https://jbi.global). Any disagreement was resolved by a third reviewer. All reviewers were the authors of the present study.

## 3 Results

### 3.1 Overview of the Studies

**Figure 2:**
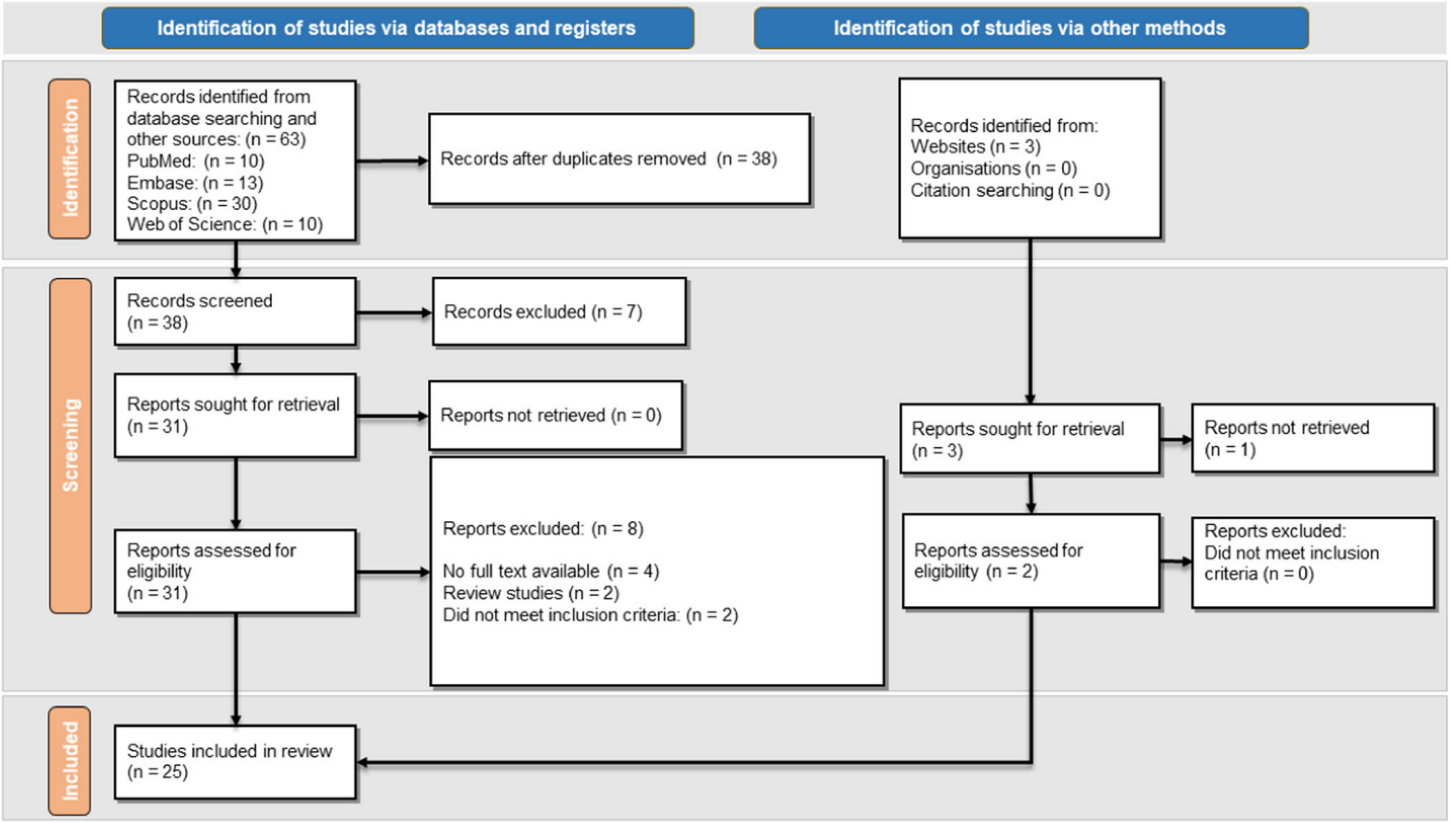
PRISMA flowchart of the literature search and selection of the articles.

The comprehensive search for this systematic review yielded 63 articles. The searched databases included PubMed, Scopus, Embase, and Web of Science international bibliometric databases. Thirty‐eight articles remained after removing duplicates and were enrolled for screening based on the evaluation of their titles and abstracts. A total of 34 studies were selected and their full texts were assessed, of which 31 studies were identified from database searching and three articles from website searching. Twenty‐three studies through database searching and two studies from website investigation were included in our study (Figure 2). Twenty‐six patients (18 males and 8 females) with the mean age of 52.96 years (± 15.64) (range: 15–85) were evaluated in the included studies. The baseline characteristics of our included studies are presented in Table 1. The results of the quality assessment of the studies is presented in Table S1.

**Table 1:** Characteristics of included studies.

ID	First author	Gender	Age	Type of cancer	Cancer treatment	Duration of treatment	Start date of dermatologic reaction	Region of dermatologic reaction	Treatment for lesions	Histopathological findings	Outcome
1	Akyurek	Male	52	Non‐small cell lung cancer	Chemotherapy with carboplatin and vinorelbin	Two chemotherapy episodes	2 weeks after the first episode, then 2 days after the second episode	Painful erythematous lesions spreading to the right arm in first episode and painful erythematous lesions on the dorsal of the left hand, extending to the left forearm in the second episode	Topical treatment in first episode and second episode, topical steroids, boric acid, and nonsteroidal anti‐inflammatory therapy in second episode	Loss of superficial epidermis, focal edema, perivascular, and dermal lymphocytic infiltration and scant extravasated erythrocytes in the biopsy sample taken on the fifth fay after the second infusion	Amelioration of erythematous and bullous lesions in the hyperpigmented regions within a few days
2	Aydogan	Male	47	Squamous cell carcinoma of the lung	Chemotherapy cisplatin and gemcitabine plus intravenous docetaxel as a second‐line therapy	Two episodes	Two days after first infusion of docetaxel	Anterior aspect of right forearm and distal arm	None	On the 15th day after the initial infusion, a punch biopsy was performed from a hyperpigmented supravenous streak. It revealed an atrophic epidermis showing hyperkeratosis, a slight vacuolization of the basal layer, necrotic keratinocytes, and diffuse basilar hyperpigmentation. In the upper dermis, lymphoplasmocytic infiltration around the dilated vessels was noted	A decrease in hyperpigmentation during the following weeks. Hyperpigmented streaks were still present 2 months after the first infusion of docetaxel. The eruption had cleared completely at the end of the sixth month
3	Chan	Male	61	Inoperable esophageal squamous‐cell carcinoma	Cisplatin and fluorouracil alongside radiotherapy. Then shifted to weekly high‐dose fluorouracil and leucovorin	Eight weeks	One day after the eighth episode	Left forearm	None	N/A	Lesions faded 2 months after the diagnosis
4	Das	Male	72	Left breast infiltrating duct carcinoma	Docetaxel, cyclophosphamide, and doxorubicin	Three times weekly for 6 weeks	After the first infusion of docetaxel	Left lower limb	Topical steroids and emollients	Basal layer degeneration, melanophages, pigment Incontinence, focal band‐like Infiltrate, and perivascular mononuclear infiltrate	N/A
5	Geddes	Female	45	Multifocal invasive ductal carcinoma of right breast	Paclitaxel, 5‐l, doxorubicin, fluorouracil, and cyclophosphamide	Twelve weekly cycles of paclitaxel and subsequently 4 cycles, every 3 weeks, of 5‐FU, doxorubicin, and cyclophosphamide	After receiving therapy	Left arm	None	N/A	The hyperpigmentation remained unchanged
6	Ghosh	Female	45	Recurrent breast carcinoma	Docetaxel every 21 days	One dose	One day after the first dose	Left forearm	Daily mometasone furoate ointment for 2 weeks	Histopathological examination at the seventh day revealed a Basketweave pattern of hyperkeratosis and spongiosis with lymphocytic and neutrophilic exocytosis, focal basal cell degeneration, papillary dermal edema irregular acanthosis, and upper dermal bandlike, perivascular lymphomononuclear infiltrates	Improvement within a week
7	Hrushesky	Male	56	Stage D prostate carcinoma	Weekly fluorouracil	Twenty‐four consecutive weeks	N/A	Left upper extremity	N/A	N/A	N/A
8	Jain	Male	30	Rectal adenocarcinoma	5‐FU	Three cycles	After two cycles of receiving 5‐FU	Overlying the right cephalic, median cubital veins, basilic, and median veins of forearm	None	Increase in the melanin incontinence and epidermal melanin content into the dermis	Pigmentation disappeared and may not warrant discontinuation of the drug
9	Jamalpur	Female	30	Breast cancer	Docetaxel	One dose	After the first docetaxel infusion	Right forearm	N/A	N/A	N/A
10	Lancman	Male	50	Diffuse large B cell lymphoma	Cyclophosphamide	One cycle	Ten days after receiving first dose of R‐Chop, and 6 days after receiving chemotherapy	Right hand	Supportive therapy	N/A	The lesions in his right arm had improved significantly after 4 months, but he developed new lesions appeared in the left arm due to changed infusion site
11	Maalouf	Female	58	Metastatic uterine leiomyosarcoma	Gemcitabine and docetaxel	N/A	After 6 weeks of chemotherapy	Arms	Changing the infusion access to a central venous catheter	N/A	The rash had abated 2 months later
12	Marcoux	Male	15	Left paratesticular embryonal rhabdomyosarcoma	Actinomycin and vincristine	Two episodes	A few hours after the first episode	Left forearm and after a single injection of actinomycin in the right arm	N/A	Disclosed discrete epidermal changes with slight vacuolization of the basal layer plus some hyperpigmentation. Rare melanophages in the superficial dermis, polymorphonuclear inflammatory infiltrate, mixed perieccrine lymphohistiocytic, and squamous metaplasia of the eccrine ducts in the biopsy sample taken on the 10th day	A decrease in the color intensity of the eruption of the left forearm and arm after 14 days. The hyperpigmented area was present after 10 months
13	Marongiu	Male	85	Kaposi's sarcoma	Vinorelbine	One dose	One day after the first infusion	N/A	N/A	N/A	N/A
14	Narayan	Male	44	Poorly differentiated carcinoma of the stomach	5‐FU	One dose	Six days after the first infusion	Right hand	None	N/A	Lesion started resolving and evolving into hyperpigmented streaks plus mild scaling in the fourth week
15	Noori	Male	52	Pulmonary adenocarcinoma	Cisplatin and pemetrexed	One dose through the left forearm and one dose through the right forearm	After the second cycle	Hyperpigmentation on the right arm and darkness on the left arm	Discontinuation of cisplatin and insertion of a chest catheter for further infusions	N/A	N/A
16	Pujol	Male	47	Grade II squamous cell carcinoma of the hypopharynx	Cisplatin and dexamethasone as well as 5‐FU	Five days	Four days after the treatment	Both upper extremities	None	A prominent vacuolar alteration of basal cells, pigment incontinence, perivascular lymphocytic infiltrate, and necrotic keratinocytes in the upper dermis	The lesions improved and were clear after 3 months
17	Rao	Male	38	Sigmoid colon adenocarcinoma	5‐FU	Four cycles	After the fourth cycle	Right and left forearms	None	N/A	Pigmentation started fading after 4 weeks
18	Suvirya	Female	42	Carcinoma of the sigmoid colon	5‐FU	Two cycles	Ten days after the second cycle	Right and left forearms	Topical hydroquinone and clobetasol propionate	Histopathological examination on the fourth day revealed sparse superficial perivascular infiltrate of melanophages with a few lymphocytes, slight flattening of rete ridges and mild fibroplasia in papillary dermis	A significant decrease in hyperpigmentation after 4 months
19	Umemura	Male	54	Non‐small cell lung cancer	Docetaxel	One dose	Ten days after the first dose	Distal left forearm	Topical corticosteroid	N/A	The lesions re‐occured 15 days after the fourth infusion
20	Yetut	Male	54	Lung adenocarcinoma	Cisplatin, pemetrexed, and vinorelbine	Twelve weeks	After the 12th week	Left forearm	Topical medication	N/A	Lesions regressed completely
21	Sarayama	Male	68	Metastatic extramammary Paget carcinoma	Docetaxel	One course	One month after the first dose of docetaxel	Anterior forearm	N/A	N/A	N/A
22	Chaiyakul	Male	66	Advanced‐stage lung cancer	Docetaxel	One cycle	Three days after the first cycle	Right foot	Topical betamethasne valerate cream	Superficial interface dermatitis consistent with supravenous serpentine dermatitis	N/A
23	Perez	Female	53	Breast carcinoma	Docetaxel	N/A	Six days after last infusion	Back of the left hand	Oral dexamethasone and cetirizine	N/A	The lesion resolved 2 weeks later with hyperpigmentation
24	Fernandes	Female/female	68, 65	Breast carcinoma	Docetaxel and trastuzumab/docetaxel and filgrastin	Two cycles/two cycles	A few days after the chemotherapy	Left forearm	Methylprednisolone	N/A	Residual hyperpigmentation/complete remission
25	Miyamoto	Male	80	Non‐small cell lung cancer	Docetaxel	Three cycles	After third infusion	Left and right forearm	Changing the infusion site	N/A	N/A

### 3.2 Different Types of Cancer

A total of 13 different cancers were reported in the included studies, including lung cancer with the most articles (*n* = 7) [17, 18, 19, 20, 21, 22, 23], breast cancer (*n* = 6) [9, 12, 24, 25, 26, 27], sigmoid carcinoma (*n* = 2) [28, 29], esophageal carcinoma (*n* = 1) [30], rectal adenocarcinoma (*n* = 1) [31], carcinoma of the stomach (*n* = 1) [32], hypopharynx carcinoma (*n* = 1) [33], Kaposi's sarcoma (*n* = 1) [34], rhabdomyosarcoma (*n* = 1) [35], leiomyosarcoma (*n* = 1) [36], diffuse large B cell lymphoma (*n* = 1) [37], Paget carcinoma (*n* = 1) [38], and prostate cancer (*n* = 1) [39].

### 3.3 Region of Dermatologic Reaction

The presentation of serpentine supravenous dermatitis is reported in various region of the body in the included studies, however, the mostly reported region is forearm which is reported in 13 studies [9, 12, 18, 21, 22, 23, 27, 28, 29, 30, 31, 35, 38]. Other reported regions included arms, foots, upper extremities, lower limbs, hands, and foots, which were all close to the infusion sites.

### 3.4 Initiation of Dermatologic Reaction

Different timelines of dermatologic finding were reported in the included studies. Nine studies have reported that the dermatitis of their patients started within 1 week after administration of the first course of chemotherapy [12, 18, 19, 24, 32, 33, 34, 35, 37], while four studies have reported that the dermatological manifestations of their patients appeared after 1 week of the first infusion of chemotherapeutic agents [9, 17, 21, 38]. In the other studies, the onset of dermatitis occurred after the second or later cycles of chemotherapy (Table 1) [7, 8, 20, 22, 23, 26, 27, 28, 29, 30, 31, 36].

### 3.5 Chemotherapeutic Agents

Multiple chemotherapeutic agents have been reported that result into serpentine supravenous dermatitis independently or with combination to other agents, including docetacel, fluorouracil, cisplatin, vinorelbin, gemcitabine, leucovorin, paclitaxel, cyclophosphamide, doxorubicin, vincristine, actinomycin, and premetrexed.

#### 3.5.1 Docetaxel

Docetaxel inhibits the synthesis of protein, DNA, and RNA and is one of the most used chemotherapeutic agents in different types of cancers in our included studies, including breast cancer and non‐small cell lung cancer. Docetaxel has been used in a total of 11 articles in this study. Docetaxel has independently induced serpentine supravenous dermatitis in seven studies [9, 12, 19, 21, 23, 26, 38] and in combination to other agents, including cisplatin, gemcitabine, cyclophosphamide, doxorubicin, trastuzumab, and filgrastin in four studies [18, 24, 27, 36].

#### 3.5.2 Fluorouracil

Fluorouracil or 5‐FU inhibits DNA synthesis and is being used as an efficient chemotherapeutic agent in the treatment of cancers. Fluorouracil has been led into serpentine supravenous dermatitis as a single therapeutic agent in five studies [28, 29, 31, 32, 39] and in combination to leucovorin, paclitaxel, doxorubicin, cyclophosphamide, and cisplatin in three of our included studies [25, 30, 33].

#### 3.5.3 Cisplatin

Cisplatin has been used for the treatment of various types of cancers, such as cervical cancer, esophageal cancer, lung cancer, and bladder neoplasms [40]. No study has been reported that single therapy with cisplatin resulted into serpentine supravenous dermatitis, however, its combination with other drugs, including gemcitabine [18], premetrexed [20], premetrexed plus vinorelbine [22], and fluorouracil [33] led to this dermatologic reaction.

#### 3.5.4 Vinorelbine

Vinorelbine, which is another chemotherapeutic agent, was mentioned in three of our included studies. In the study by Marongiu, Lissia, and Cottoni [34], single chemotherapy for Kaposi's sarcoma with vinorelbine resulted into serpentine supravenous dermatitis a day after the first infusion. Moreover, in two studies [17, 22], the combination of vinorelbine with premetrexed, cisplatin, and carboplatin developed the dermatologic reaction.

### 3.6 Histopathological Findings

Nine studies reported the histopathological findings of skin biopsies [12, 17, 18, 19, 24, 29, 31, 33, 35]. The detailed information of the skin biopsy findings is presented in Table 1, although, the freauently reported pathologies were dermal lymphocytic infiltration, necrotic keratinocytes, hyperkeratosis, and basal layer hyperpigmentation.

## 4 Discussion

Systemic and local treatment of cancer can cause some side effects on the skin, hair, and nails [41]. SSH is one of the side effects which was reported during chemotherapy [11]. Although SSH is usually seen due to chemotherapy agents, this phenomenon occurs due to other drugs such as minocycline and ofloxacin which are used for leprosy treatment [42]. Moreover, persistent SSH could be related to collagen vascular disease such as systemic progressive sclerosis, rheumatoid arthritis, and systemic lupus erythematous, and advanced human immunodeficiency virus (HIV) disease. In a case series, idiopathic SSH was considered as a cutaneous manifestation of HIV disease [43]. SSH is an erythematous or hyperpigmented eruption on the superficial veins which is seen in the injection site during applying chemotherapeutic agents [44]. Chemotherapy agents such as 5‐FU, vincristine, vinorelbine, and docetaxel are the most common agents causing this phenomenon [45, 46]. These lesions progress within 24 h to 15 days [44]. However, this side effect is usually a self‐limited process and will be recovered after the termination of chemotherapy [11, 47] (Figure 1).

SSH, while commonly associated with intravenous chemotherapy agents, can also occur with certain oral medications, such as hydroxychloroquine [48]. This phenomenon has been reported in cases of patients taking hydroxychloroquine for rheumatoid arthritis or other rheumatologic conditions [48]. Similarly, minocycline, an oral tetracycline antibiotic used for acne and other indications, has been implicated in causing hyperpigmentation [49].

The proposed mechanisms behind this reaction are similar to those seen with chemotherapeutic agents—the drugs may cause inflammation, vascular damage, and extravasation, leading to pigment deposition along the venous pathways [50]. However, unlike chemotherapy‐induced cases which are often self‐limiting, serpentine hyperpigmentation from oral medications like hydroxychloroquine can sometimes persist even after drug discontinuation [48].

Therefore, it is important to recognize that this distinctive pigmentary dermatitis can occur not just with intravenous drug administration, but also with certain oral medications. Clinicians should remain vigilant for this potential side effect when prescribing drugs like hydroxychloroquine or minocycline.

The pathogenesis of this phenomenon has not been exactly identified, but there are some hypotheses. Drug toxicity may lead to extravasation injury and stimulate melanin synthesis [51]. Endothelium damage can cause venous insufficiency and chemotherapy agents leak through the damaged endothelium [52, 53]. Therefore, the cytotoxicity of these agents can damage directly to basal keratinocytes and melanocytes due to the penetrating of these agents through the damaged endothelium [35, 53] (Figure 1).

In this article, a systematic review of the case reports was conducted, including 25 articles. This is the first systematic review of the SSH caused by the chemotherapy drugs. Twenty‐six patients were considered.

In this study, the most prevalent cancer in males was lung cancer and the most prevalent cancer in females was breast cancer. Due to the overview of the cancer epidemiology reported in 2023, breast cancer is reported as the most common cancer among females. Although lung cancer is the third‐ranked cancer among females, it is known as the common cancer in the male population [54].

In this review study, the most type of cancer reported during this phenomenon was lung cancer. All seven patients who had suffered from lung cancer were male. Three patients with non‐small cell lung cancer, two patients with lung adenocarcinoma, one patient with squamous cell carcinoma of lung, and one patient with advanced‐stage lung cancer were considered in this study [11, 20, 21, 44, 55, 56, 57]. Six patients with breast cancer were reported in this study and five patients were female [7, 26, 27, 51, 52, 58].

SSH was seen in other cancers such as sigmoid cancer (two patients), esophageal cancer, rectal adenocarcinoma, carcinoma of the stomach, hypopharynx carcinoma, Kaposi sarcoma, rhabdomyosarcoma, liomyosarcoma, diffuse large B cell lymphoma, paget carcinoma, and prostate cancer.

In both patients with sigmoid cancer, 5‐FU was used as chemotherapy agent. In Suvirya's study, a 42‐year‐old‐woman with sigmoid cancer had taken the drug through peripheral route [29]. Hyperpigmentation was seen in both upper extremities. Multiple brownish macules appeared on both her arms. Both soles were involved [59]. In Rao's study, a 38‐year‐old man who had suffered from sigmoid adenocarcinoma was reported [28]. Hyperpigmentation was appeared in his both forearm following applying 5‐FU. Hyperpigmentation was usually seen in sun‐exposed regions [60].

Of the 26 patients, 11 patients had docetaxel in the chemotherapy regime, eight patients had 5‐FU in chemotherapy regime, and nine patients applied another drugs.

Moreover, capecitabine, doxorubicin, cyclophosphamide, and especially 5‐FU could cause hyperpigmentation in other places such as tongue, palm, and soles besides the injection site through the upregulating the activity of melanocyte‐stimulating hormone [61]. Although topical 5‐FU is applied in the treatment of vitiligo through its antimitotic activity, usage of this drug systematically could make hyperpigmentation on extremities and tongue [62, 63, 64]. When 5‐FU are used systematically, it destroys keratinocytes and proliferates melanocytes. So all these mechanisms could lead to cause hyperpigmentation lesions [65, 66].

Of 26 patients with these lesions, one patient was detected with HIV disease in the past medical history. In Lancman's study, a 50‐year‐old man with HIV and DLBCL who had taken R‐CHOP as chemotherapy treatment. His reaction was appeared on his right hand 6 days after applying chemotherapy agents [37]. Although SSH is seen in patients with HIV, these lesions in this patients are usually bilateral and are not related to the infusions [43].

Of the 26 patients, seven cases used topical steroids as the treatment of this phenomenon, one case used oral dexamethasone and cetirizine to treat this lesion, two cases used methylprednisolone pulse, three cases changed the route of chemotherapy administration, and 12 cases did not use any treatment for this lesion. In three cases, lesions still remained. In one of the patients who was treated with methylprednisolone pulse, the lesion was not resolved. One of the patients with residual hyperpigmentation was used topical steroids, boric acid, and oral anti‐inflammatory therapy and another patient with residual lesion did not apply any treatments.

Regarding second‐line medications, in the case reported by Aydogan et al. [18], the patient with squamous cell carcinoma of the lung received docetaxel as a second‐line therapy after being treated with cisplatin and gemcitabine initially. This docetaxel treatment led to the development of persistent SSH.

As for cases where the lesions remained, the manuscript mentions three such instances: In Akyurek et al.'s case [Case 1] [17], the patient experienced amelioration of erythematous and bullous lesions within days, but the hyperpigmented regions persisted. No specific timeframe for resolution was mentioned. In Lancman et al.'s case [37], involving a patient with HIV and diffuse large B‐cell lymphoma, the lesions on the right arm improved significantly after 4 months. However, new lesions appeared on the left arm due to change in the infusion site. In Fernandes et al.'s case [27], with two breast cancer patients, one patient had residual hyperpigmentation even after treatment with methylprednisolone, while the other achieved complete remission. No timeframe was specified.

Nine patients had histopathology report. In histopathological study of the skin biopsy, loss of superfacial epidermis, dermal lymphocytic infiltration, extravasated erythrocytes, atrophic epidermis with hyperkeratosis, necrotic keratinocytes, layer degeneration, pigment incontinence, focal band‐like, and an increase in the epidermal melanin content and melanin incontinence into the dermis were reported [11, 33, 35, 44, 52, 55, 58, 59, 67].

In summary, in this review article 26 patients with different types of cancer and treatments who had shown serpentine supravenous hyperpigmented eruption as a side effect of chemotherapy were studied. Lung cancer and breast cancer were the most common cancers reported. Docetaxel and 5‐FU were the most common agents. There is no need skin biopsy to diagnosis this phenomenon. Most of patients used topical and supportive therapy for the treatment of this phenomenon. Although it was improved in most cases, in few cases it did not recover. In the end, usage of topical treatment and changing the route of drug administration such as central venous line can help the health staffs to manage this phenomenon. They should know that these lesions could be self‐limited and in the most cases there is no need for the treatment.

## 5 Conclusion

Serpentine supravenous hyperpigmented eruption is one of the dermatological side effect of chemotherapy agents. It is self‐limited disease but in few cases it did not improve. Therefore, the acknowledgment of the pathophysiology and management in different patients, in conjunction with the chemotherapy agents been used, is essential. However, the main management for this phenomenon is changing the peripheral access to a central access for infusion of chemotherapeutic agents.

## Author Contributions

**Hanieh Radkhah:** conceptualization, writing–review and editing, project administration, supervision, visualization. **Saba Maleki:** investigation, writing–original draft, validation, visualization, resources. **Razman Arabzadeh Bahri:** investigation, writing–original draft, writing–review and editing, methodology, data curation, formal analysis, resources.

## Ethics Statement

The authors have nothing to report.

## Conflicts of Interest

The authors declare no conflicts of interest.

## Transparency Statement

The lead author Razman Arabzadeh Bahri affirms that this manuscript is an honest, accurate, and transparent account of the study being reported; that no important aspects of the study have been omitted; and that any discrepancies from the study as planned (and, if relevant, registered) have been explained.

## Supporting information

Supporting information.

## Data Availability

All relevant data are within the manuscript.
