# Supplementary material for: Serpentine Supravenous Hyperpigmentation, a Phenomenon Following the Administration of Chemotherapeutic Agents: A Systematic Review
Source: Health Sci Rep. 2024 Dec 19;7(12):e70294. doi: 10.1002/hsr2.70294 (PMC11659118; doi:10.1002/hsr2.70294)
Supplement: Supplementary file 1 — Supporting information. [file HSR2-7-e70294-s001.docx]

| ID | First Author | Patient’s demographic | Patient’s history | Clinical condition | Assessment methods | Intervention | Post-intervention | Adverse events | Takeaway lessons |
| --- | --- | --- | --- | --- | --- | --- | --- | --- | --- |
| 1 | Akyurek | Yes | Yes | Yes | Yes | Yes | Yes | No | Yes |
| 2 | Aydogan | Yes | Yes | Yes | Yes | No | Yes | No | Yes |
| 3 | Chan | Yes | Yes | Yes | Unclear | No | Yes | No | Yes |
| 4 | Das | Yes | Yes | Yes | Yes | Yes | Unclear | No | Unclear |
| 5 | Geddes | Yes | Yes | Yes | Unclear | No | Yes | No | Yes |
| 6 | Ghosh | Yes | Yes | Yes | Yes | Yes | Yes | No | Yes |
| 7 | Hrushesky | Yes | Yes | Yes | Unclear | Unclear | Unclear | No | Unclear |
| 8 | Jain | Yes | Yes | Yes | Yes | No | Yes | No | Yes |
| 9 | Jamalpur | Yes | Yes | Yes | Unclear | Unclear | Unclear | No | Unclear |
| 10 | Lancman | Yes | Yes | Yes | Unclear | Yes | Yes | No | Yes |
| 11 | Maalouf | Yes | Yes | Yes | Unclear | Yes | Yes | No | Yes |
| 12 | Marcoux | Yes | Yes | Yes | Yes | Unclear | Yes | No | Yes |
| 13 | Marongiu | Yes | Yes | Yes | Unclear | Unclear | Unclear | No | Unclear |
| 14 | Narayan | Yes | Yes | Yes | Unclear | No | Yes | No | Yes |
| 15 | Noori | Yes | Yes | Yes | Unclear | Yes | Unclear | No | Unclear |
| 16 | Pujol | Yes | Yes | Yes | Yes | No | Yes | No | Yes |
| 17 | Rao | Yes | Yes | Yes | Unclear | No | Yes | No | Yes |
| 18 | Suvirya | Yes | Yes | Yes | Yes | Yes | Yes | No | Unclear |
| 19 | Umemura | Yes | Yes | Yes | Unclear | Yes | Yes | No | Yes |
| 20 | Yetut | Yes | Yes | Yes | Unclear | Yes | Yes | No | Unclear |
| 21 | Sarayama | Yes | Yes | Yes | Unclear | Unclear | Unclear | No | Unclear |
| 22 | Chaiyakul | Yes | Yes | Yes | Yes | Yes | Unclear | No | Yes |
| 23 | Perez | Yes | Yes | Yes | Unclear | Yes | Yes | No | Yes |
| 24 | Fernandes | Yes | Yes | Yes | Unclear | Yes | Yes | No | Yes |
| 25 | Miyamoto | Yes | Yes | Yes | Unclear | Yes | Unclear | No | Unclear |

Table S1. The results of the quality assessment of the included studies
